# Structural diversity and biological significance of glycosphingolipids in pathogenic and opportunistic fungi

**DOI:** 10.3389/fcimb.2014.00138

**Published:** 2014-09-25

**Authors:** Luciana L. Guimarães, Marcos S. Toledo, Felipe A. S. Ferreira, Anita H. Straus, Helio K. Takahashi

**Affiliations:** ^1^Laboratory of Glycoconjugate Immunochemistry, Department of Biochemistry, Escola Paulista de Medicina, Universidade Federal de São PauloSão Paulo, Brazil; ^2^Laboratory of Natural Products, Department of Pharmaceutical Sciences, Universidade Santa CeciliaSantos, Brazil

**Keywords:** fungal glycosphingolipids, glucosylceramide, glycosylinositol phosphorylceramides, glycosphingolipid synthesis inhibitors, fungal membrane microdomains

## Abstract

Glycosphingolipids (GSLs) are ubiquitous membrane components and have key roles in biological systems, acting as second messengers or modulators of signal transduction by affecting several events, ranging from cell adhesion, cell growth, cell motility, regulation of apoptosis and cell cycle. Over the last 20 years our laboratory and other research groups determined the glycan and ceramide structures of more than 20 GSLs from several pathogenic/opportunistic fungi, using a combination of gas chromatography, mass spectrometry, nuclear magnetic resonance as well as other immunochemical and biochemical techniques. Fungal GSLs can be divided in two major classes: neutral GSLs, galactosyl- and glucosylceramide (GlcCer), and acidic GSLs, the glycosylinositol-phosphorylceramides (GIPCs). Glycosyl structures in fungal GIPCs exhibited significant structural diversity and distinct composition when compared to mammalian GSLs, e.g., the expression of inositol-mannose and inositol-glucosamine cores and the terminal residue of β-D-galactofuranose which are absent in mammalian cells. Studies performed by our group demonstrated that GIPC (Gal*f*β 6[Manα3]Manα2InsPCer) elicited in patients with paracoccidioidomycosis an immune response with production of antibodies directed to the terminal residue of β-D-galactofuranose. Further studies also showed that inhibition of GlcCer biosynthetic pathways affects fungal colony formation, spore germination and hyphal growth, indicating that enzymes involved in GlcCer biosynthesis may represent promising targets for the therapy of fungal infections. Recently, it was shown that GlcCer and GIPCs are preferentially localized in membrane microdomains and monoclonal antibodies directed to these GSLs interfere in several fungal biological processes such as growth and morphological transition. This review focuses on glycan structures carried on sphingolipids of pathogenic/opportunistic fungi, and aspects of their biological significance are discussed.

## Introduction

Glycosphingolipids (GSLs) are ubiquitous membrane components present mostly in the outer leaflet of the plasma membrane with their carbohydrate head groups exposed to the extracellular space, and mainly organized in microdomains by association with sterols and specific proteins.

**Figure 1:**
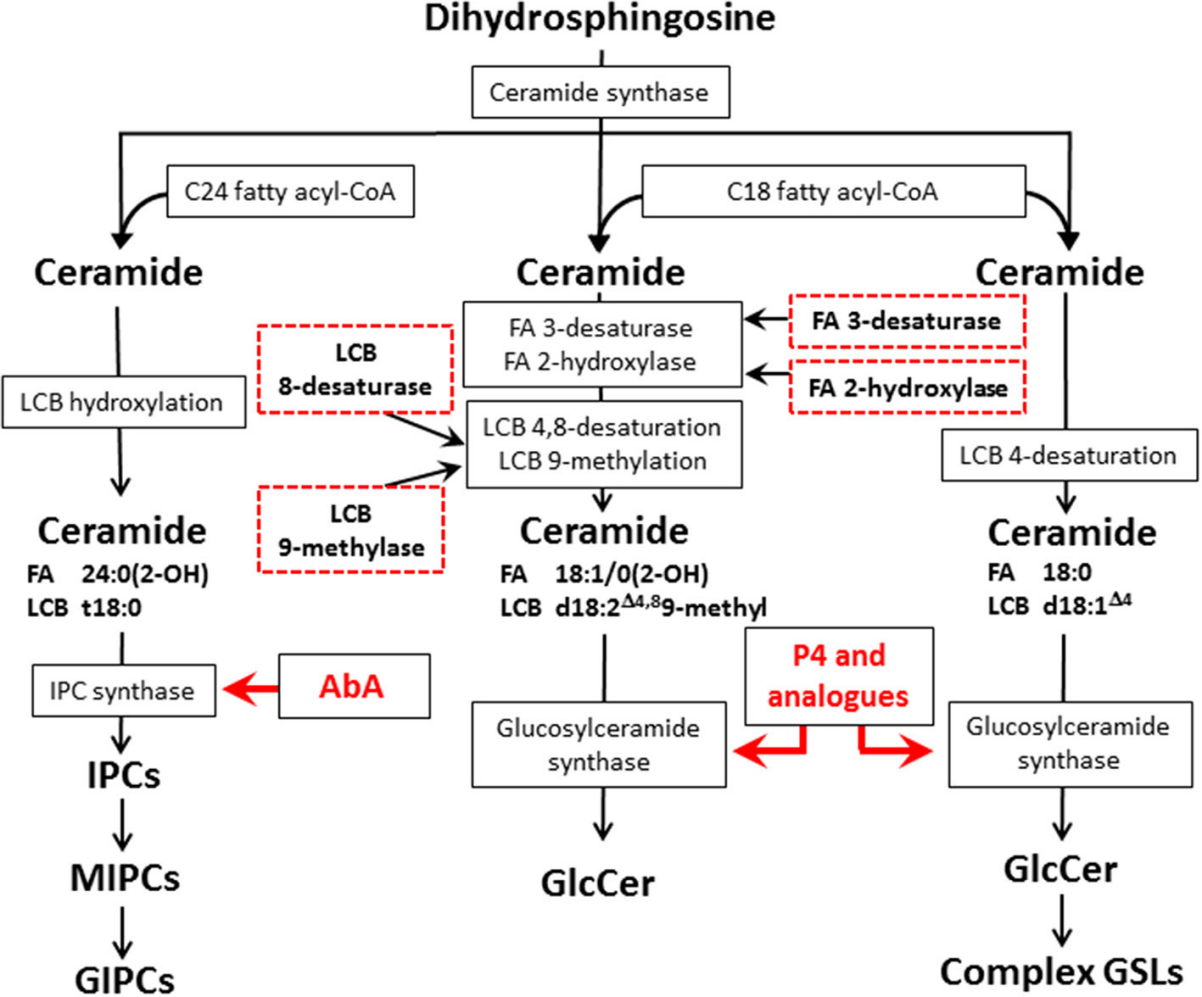
**Fungal and mammal GSLs biosynthetic pathways and potential targets for new antifungal therapies**. The scheme displays the biosynthetic pathways of fungal GIPCs (left), fungal GlcCer (center), and mammalian GSLs (right). The red arrows indicates the biosynthetic step where the inhibition by AbA and P4/analogs occurs, the red dashed boxes in the glycosphingolipid biosynthetic pathway indicate potential targets for the development of new antifungal therapies. LCB, long chain base; FA, fatty acid; IPC, inositol phosphorylceramide; MIPC, mannosylinositol phosphorylceramide; GIPC, glycosylinositol phosphorylceramide; AbA, Aureobasidin A; P4, D-threo-1-phenyl-2-palmitoyl-3-pyrrolidinopropanol.

The glycosphingolipid biosynthesis starts by the action of serine palmitoyltransferase which condensates palmitoyl-CoA with serine forming 3-keto-sphinganine, the keto group is reduced to hydroxyl group thus forming the dihydrosphingosine (also termed sphinganine), at this point, as shown in Figure 1, the dihydrosphingosine may be hydroxylated on the C4 to form the phytosphingosine (4-hydroxysphinganine). Specific acyl transferases act upon dihydrosphingosine and phytosphingosine forming dihydroceramide or phytoceramide, respectively. In dihydroceramides, a desaturase inserts a double bound at C4 converting it in ceramide (Rhome et al., 2007). In fungi Glc or Gal residues are transferred to ceramide in the assembly of GlcCer or GalCer whereas the more complex glycan moieties of GIPCs are built up on phytoceramide (Takahashi et al., 2009). After glycosylation, GSLs are transported to the outer leaflet of the cell membrane forming membrane microdomains, also as described by Longo et al. (2013), GlcCer may also be found in lipid vesicles in the cell wall, probably being part of the exocytosis transport system.

Studies analyzing the roles of glycosphingolipids (GSLs) in different biological systems have demonstrated their association with: (i) oncogenic transformation; (ii) embryonic development; (iii) control of cell division; (iv) cell adhesion and motility; (v) signal transduction pathways by glycosinapses, e.g., carbohydrate-carbohydrate interactions; and (vi) control of cell phenotype (Hakomori, 2004, 2008). Since the first description of GSLs in fungi (Steiner et al., 1969), numerous studies focused in structural elucidation and biological significance of these GSLs revealed the importance of these sphingolipids in normal fungal morphogenesis and infectivity. In the last 25 years our lab has made efforts to isolate and analyze the structure of more than 20 new fungal GSLs. In this review we focus on glycan structures carried on sphingolipids of pathogenic/opportunistic fungi and multiple aspects of their biological significance.

## Structural elucidation of GSLs isolated from pathogenic fungi

Our laboratory and other research groups have characterized a number of neutral GSLs, glucosyl- and galactosylceramide (GlcCer and GalCer), as well as acidic GSLs—the glycosylinositol-phosphorylceramides (GIPCs) from pathogenic/opportunistic fungi, using a combination of high performance thin layer chromatography (HPTLC), gas chromatography/mass spectrometry (GC/MS), single quadrupole mass spectrometry, ^1^H/^13^C nuclear magnetic resonance—Correlation Spectroscopy (COSY), Total Correlation Spectroscopy (TOCSY), Nuclear Overhauser Effect Spectroscopy (NOESY) as well as other immunochemical and biochemical techniques.

**Figure 2:**
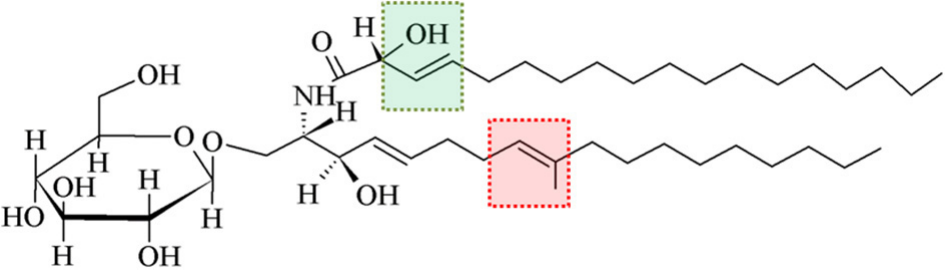
**A typical glucosylceramide from fungi**. Differently from mammalian GlcCer, fungal GlcCer displays structural peculiarities in their ceramide, such as presence of (E)-Δ^8^-unsaturation and a methyl group linked to sphingosine (highlighted by red box). Another feature that is particular to fungal GlcCer is the (E)-Δ^3^-unsaturation and the presence of a hydroxyl group linked to C2 of the fatty acid moiety (highlighted by green box) (Toledo et al., 1999).

As shown in Figure 2 contrasting to mammalian monohexosilceramides, fungal GlcCer and GalCer displays interesting structural peculiarities in their ceramide moiety, such as the presence of (E)-Δ^8^-unsaturation and a methyl group linked to sphingosine, i.e., (4E,8E)-9-methyl-4,8-sphingadienine as long chain base (d19:2), In *Cryptococcus neoformans*, the methylation in C9 on the sphingosine backbone of GlcCer is associated with fungi virulence, since the mutation of the sphingolipid C9 methyltransferase gene (SMT1), resulted in loss of more than 80% of its virulence, when compared to the wild-type and/or the reconstituted strains (Singh et al., 2012).

Another feature that is particular to fungal GlcCer/GalCer is the (E)-Δ^3^-unsaturation and the presence of a hydroxyl group linked to C2 of the fatty acid moiety, having either N-2′-hydroxyoctadecanoate (h18:0) or the unsaturated form N-2′-hydroxy-(E)-3′-octadecenoate (h18:1) [(E)-13-unsaturated fatty acid] (Takahashi et al., 2009). GlcCer from mycelium forms of *Paracoccidioides brasiliensis* and *Histoplasma capsulatum* present a higher percentage of unsaturated fatty acids, indicating that the temperature change which induces the transition of mycelium to yeast forms possibly activates a fatty acid desaturase (Toledo et al., 1999, 2001).

Additionally, for yeast forms of *Sporothrix schenckii* it was observed that the expression of both GlcCer and GalCer was approximately equimolar, while mycelial forms displayed only GlcCer. These differences in neutral GSLs expression suggest that the activation of GalCer synthase may accompanies the mycelium to yeast transition, or, conversely, the suppression of this activity may accompany the yeast to mycelium transition in this fungus (Toledo et al., 2000). Concurrently in two non-dimorphic fungi *Aspergillus fumigatus* and *Aspergillus niger*, it was reported the expression of both GlcCer and GalCer, with GalCer bearing a high percentage of unsaturated fatty acid when compared to GlcCer (Villas Boas et al., 1994; Toledo et al., 1999; Levery et al., 2000).

**Figure 3:**
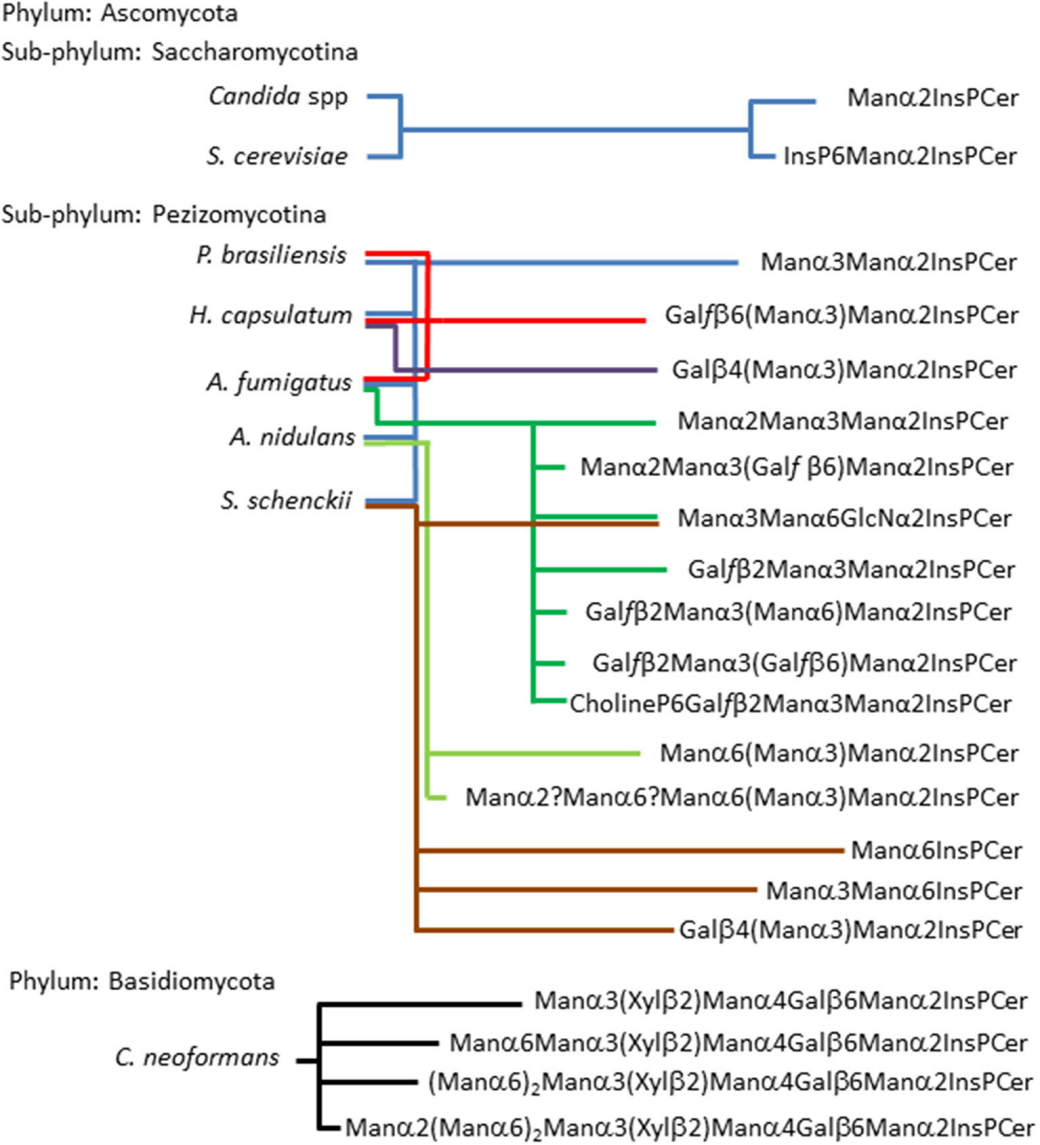
**Acidic glycosphingolipids found in fungi according to fungal phylogenetic relationships**. The scheme shows two sub-phylum from the Phylum Ascomycota: sub-phylum Saccharomycotina and sub-phylum Pezizomycotina. The former is represented by two of the most studies yeast, *Candida spp* and *S. cerevisiae*, their GIPCs were described by Steiner (Steiner et al., 1969), as two basic structures (Man2IPC and IP6Man2IPC). Five fungi are representing the sub-phylum Pezizomycotina, as can be observed they present the most variable repertoire of glycan structures in three possible core: Manα2IPC, which is present in all five fungi; GlcNα2IPC, which is present in *A. fumigatus* and *S. schenckii*; and Manα6IPC which is present only in *S. schenckii* (Barr and Lester, 1984; Barr et al., 1984; Toledo et al., 1995, 2000, 2007; Levery et al., 1996, 1998; Loureiro y Penha et al., 2001; Aoki et al., 2004; Suzuki et al., 2008). Phylum Basidiomycota is represented by *C. neoformans* which presents GIPCs with up to eight residues of carbohydrates expressing xylose residues. It is noteworthy that its capsule is rich in xylose (Heise et al., 2002).

On the other hand, inositol phosphorylceramides (IPCs) and their glycosylated derivatives (GIPCs) are widely distributed among fungal species of the two phyla Ascomycota and Basidiomycota. Fungal GIPCs, display a significant glycosyl structural variation (Barr and Lester, 1984; Barr et al., 1984; Levery et al., 1996, 1998; Loureiro y Penha et al., 2001; Toledo et al., 2007; Suzuki et al., 2008). The glycan structures of fungal GIPCs are built-up on three different “cores”: (i) GlcNα1-2Ins; (ii)Manα1-6Ins; and (iii) Manα1-2Ins. The glycosylation of IPCs seems to occur upon a ceramide mainly composed by t18:0 4-hydroxysphinganine (phytosphingosine) and h24:0 fatty acid (Takahashi et al., 2009). It is worth mentioning that the lipid moiety of these GIPCs when compared to glucosyl- and galactosylceramide displayed different sphingosines, with 4,8-diene-9-methyl-sphingo base as precursor for GlcCer/GalCer synthesis, and phytosphingosine for IPC synthesis, suggesting a dichotomy in the biosynthetic pathway of fungal neutral and acidic GSLs (Leipelt et al., 2001). The expression of GIPCs in fungi is summarized in Figure 3.

From an evolutionary perspective, an analysis of GSLs in basal lineages of fungi, such as zygomycetes (James et al., 2006; McLaughlin et al., 2009), revealed that this phylum expresses only neutral GSLs, no inositol-containing sphingolipids were detected (Aoki et al., 2004), differently from higher fungi representatives such as Ascomycota and Basidiomycota where both neutral GSLs and GIPCs are expressed.

Thus, considering the structural diversity of fungal GSLs, studies aiming to investigate the structure-function relationship of these glycoconjugates and their phylogenetic distribution in fungi kingdom may open new perspectives allowing to identify specific targets for new generation of antifungal drugs.

## Inhibition of GSL biosynthetic pathways as targets for new antifungal therapies

Hence, in order to better understand the importance/biological role of GSLs in different fungi, combined with studies searching for new alternatives for antifungal therapies, a series of studies were conducted in our laboratory to analyze the inhibition effect of key enzymes involved in biosynthetic pathways of fungal GlcCer and GIPCs.

Studies performed with inhibitors of GlcCer synthase, D-threo-1-phenyl-2-palmitoyl-3-pyrrolidinopropanol (P4) and D-threo-3P,4P-ethylenedioxy-P4 (EDO-P4), showed a strong inhibition of germination and hyphal growth, affecting also fungal colony formation of *A. fumigatus* and *A. nidulans* (Levery et al., 2002). Similar results were observed when *P. brasiliensis, H. capsulatum, S. schenckii*, and *C. neoformans* were cultivated in the presence of P4 (Takahashi et al., 2009). It is worth mentioning that antimicrobial peptides, such as the plant defensin RsAFP2, also display antifungal activity against *Candida* isolates by interaction with fungal GlcCer (Tavares et al., 2008; Thevissen et al., 2012; Silva et al., 2014). Further improvement of existing GlcCer synthase inhibitors, based on the active site of the fungal enzyme may confer higher selectivity for these compounds, a key step for a more efficient therapy of fungal infections, with fewer side effects on the patients.

Other approaches may also lead to interesting results in studies regarding GlcCer and its influence in host/pathogen interactions, which consists in the use of GlcCer-deficient mutants (Δgcs1) of pathogenic fungi. As shown by Rittershaus et al. (2006) *C. neoformans* mutant strain lacking GlcCer was unable to grow *in vitro* at a neutral/alkaline pH in the presence of 5% CO_2_, a condition that mimics the host extracellular environment, such as in alveolar spaces or in the bloodstream. However, growth of these mutants was similar to wild type at acidic pH, which mimics the host intracellular environment, such as macrophage-phagolysosome. Furthermore, when these GlcCer defective mutants were incubated with J774.16 macrophage-like cells, no differences in intracellular growth of mutant cells were observed in comparison to the wild-type, suggesting that GlcCer does not have a relevant role in *C. neoformans* intracellular development. Considering the fact that in *Cryptococcus* infections they are predominantly in the extracellular environment, GlcCer may represent a highly relevant molecule associated with virulence of *C. neoformans*.

In the last few years, RNAi technology has been used in silencing genes in *Saccharomyces* spp and *Candida albicans* yeasts (Drinnenberg et al., 2009; Moazeni et al., 2012). Also, some specific features of fungal GlcCer may represent potential targets for therapy, e.g., methylation at C9 and desaturation at C8 of sphingosine, hydroxylation at C2 and desaturation at C3 of the fatty acid (Figure 1). Using a similar approach, the expression of fungal glucosylceramide synthase (GCS) as well as other enzymes related to this biosynthetic pathway could be reduced. As pointed out in a recent review by Del Poeta et al. (2014) GlcCer may be considered a key molecule in fungal infectivity, therefore, this approach may help to develop new therapeutic strategies based on silencing specific target sequences not present in mammals.

Concerning the other biosynthetic route of GSLs in fungi, the IPC and GIPCs synthesis, the first step is catalyzed by the transfer of a phosphoinositol group from a phosphatidylinositol to a ceramide (or phytoceramide) (Nagiec et al., 1997), which also represents potential target for the development of new antifungal drugs. In cultures of *Saccharomyces cerevisiae*, the inhibition of IPC synthase by Aureobasidin A (AbA), a highly specific pharmacological inhibitor of IPC synthase (Takesako et al., 1993) (Figure 1), led to the abnormal budding and fungal death (Endo et al., 1997). Takesako et al. (1993) also showed that AbA oral treatment in mice with systemic candidiasis was effective and showed low toxicity for the host. In a more recent experimental approach Tan and Tay (2013) tested the *in vitro* susceptibility of 92 clinical isolates of various *Candida* species to AbA. These authors described that planktonic Candida yeasts were more susceptible to AbA than *Candida* forms present in biofilm (MIC_50_ of 1.0 vs. 8.0 μg.mL^−1^, respectively). In this study it was also demonstrated that AbA inhibited filamentation and lead to short hyphae formation which may have disabled the biofilm development by *C. albicans*, though biofilm formation and development is a highly complex process which still remain to be fully understood (Shopova et al., 2013; Guimarães and Takahashi, 2014).

Blocking the synthesis of GIPCs with inhibitors also may confer the additional advantage of microbial selectivity considering the fact that this class of GSL is absent in mammalian cells. However, fungi that lack GIPC biosynthetic pathway, such as zygomycetes presented resistance to AbA (Aoki et al., 2004) and they will probably be resistant to others inhibitors of IPC synthase. Therefore, the use of enzymatic inhibitors for one or both GSL biosynthetic pathways must vary according to the fungi, allowing the combination of both therapies for most efficient antifungal therapies by blocking GSC and IPC synthase without affecting the biosynthetic pathways of the host.

## Fungal GSLs as modulators of host immune response

Besides the importance of GSLs for normal fungal development, studies performed by our group and other investigators have shown that some fungi elicit immune responses in the infected host. More specifically, it was demonstrated that GIPC Pb-3 (Gal*f*β 6[Manα3]Manα2InsPCer) elicited in patients with paracoccidioidomycosis (PCM) an immune response with production of antibodies directed to the terminal residue β-D-galactofuranose. Also, several GIPCs from *H. capsulatum* and *A. fumigatus* bearing a terminal residue of β Gal*f* presented cross- reactivity with sera of PCM (Barr and Lester, 1984; Toledo et al., 1995, 2007; Bertini et al., 2007). The primary immune response of patients with PCM was associated with IgM production and further switched to IgG1. IgG1 titers decreased after 5 months of antifungal therapy with sulfamethoxazole-trimethoprim accompanied with the decline of the symptoms (Bertini et al., 2007).

Passive immunization with mouse monoclonal antibody directed to *Cryptococcus* GlcCer was reported to prolong survival of mice infected with *C. neoformans* (Rodrigues et al., 2007). Conversely, data from our laboratory using a specific monoclonal antibody directed to fungal GlcCer did not show any significant inhibitory effect on *P. brasiliensis, H. capsulatum*, and *S. schenckii* colony formation units and fungal growth rate (Toledo et al., 2010). Although promising, the effect of anti-GlcCer antibodies on humans should be carefully assessed in order to fully understand the mechanisms of the modulatory response to anti-GlcCer antibodies as well as to determine their effectiveness as a therapy for different mycosis.

Concerning the cellular immune response against GSLs, experiments were carried out with concanavalin A activated BALB/c lymphonode cells and showed that purified preparations of GIPCs from *P. brasiliensis* (Manα3Manα2IPC and Gal*f*β 6[Manα3]Manα2IPC), *A. fumigatus* (Manα2Manα3[Gal*f*β 6]Manα2IPC) and *S. schenckii* (Manα3Manα6GlcNα2IPC), as well as, GlcCer and GalCer from these three fungi were able to inhibit T lymphocyte proliferation *in vitro* in a dose-dependent manner. It was observed an IC_50_ ≤ 5 μM for GIPCs, whereas an IC_50_ of 20 μM was observed for GlcCer and GalCer (Takahashi et al., 2009). Studies performed with GlcCer from *A. fumigatus* revealed that this GSL was able to activate *in vitro* mouse and human natural killer T cells (iNKT cells), and to induce airway hyperreactivity in mice (Albacker et al., 2013).

The data above indicate that fungal GSLs, presenting unique monosaccharide sequences and ceramide moieties may influence both humoral and cellular responses and potentially may open new vistas in this field.

## Fungal GSLs in membrane domains

**Figure 4:**
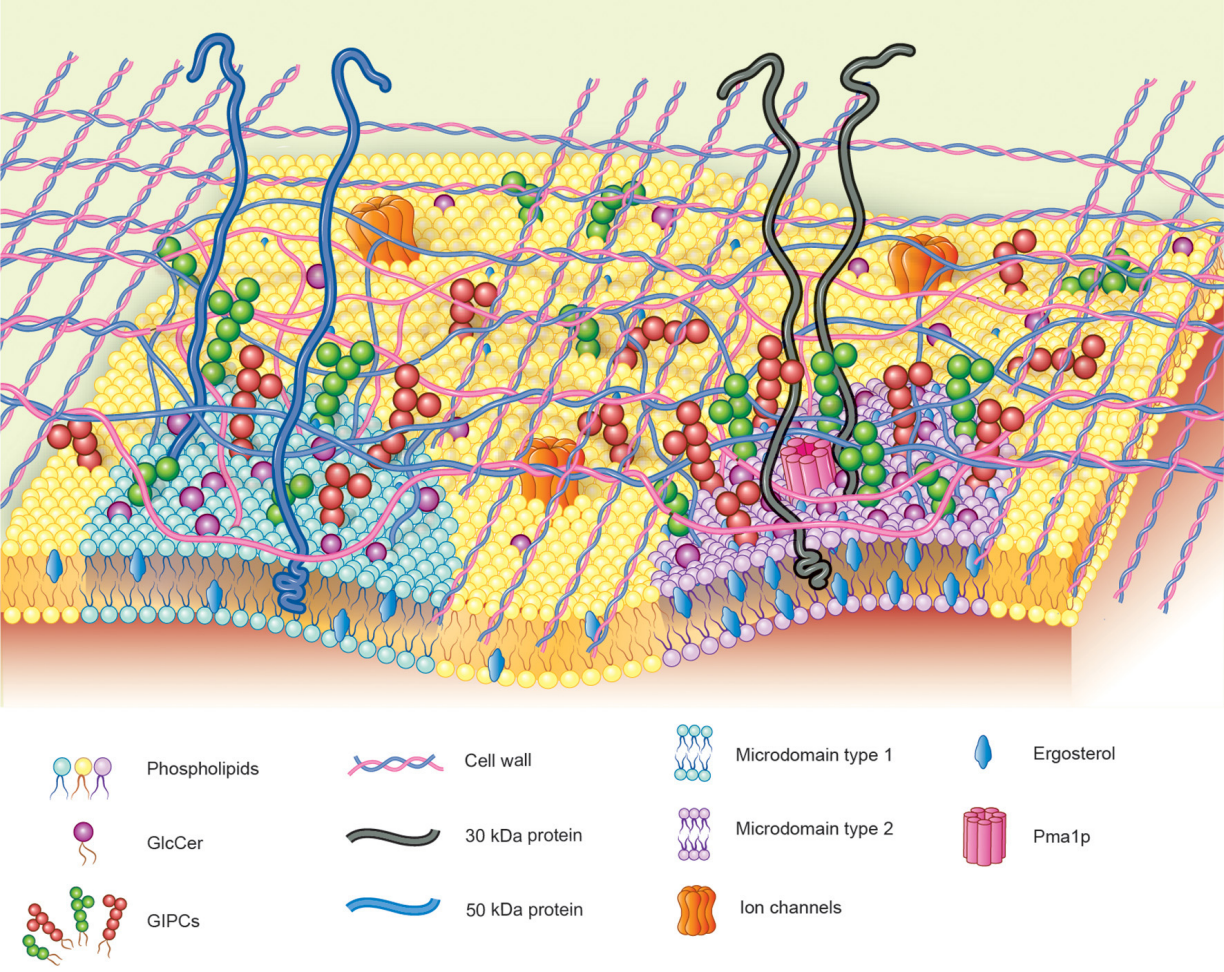
**Hypothetical model proposed for fungal membrane microdomains**. Two populations of detergent-resistant membrane microdomains are displayed in fungal plasma membrane: Type-1: ergosterol-independent microdomain, rich in integrin-like proteins and GSLs, possibly involved in signal transduction; Type-2: ergosterol-enriched microdomains containing Pma1p and the 30 kDa laminin-binding protein.

Recently it was shown by membrane microdomain isolation protocols that GlcCer and GIPCs are preferentially localized in these membrane domains. Studies performed by our group demonstrated that~40% of ergosterol from membranes of *H. capsulatum* is present in membrane microdomain fractions resistant to treatment with non-ionic detergent at 4°C (Tagliari et al., 2012). These ergosterol-enriched membrane microdomains showed a peculiar protein distribution and a distinct degree of resistance to treatment with methyl-beta-cyclodextrin (mβ CD), a sterol chelator, suggesting the existence of two populations of membrane microdomains in *H. capsulatum* yeast forms: Type 1, ergosterol-independent microdomains rich in integrin-like 50 kDa protein and GlcCer and GIPCs, possibly involved in signal transduction; and Type 2, ergosterol-dependent microdomains containing Pma1p and the 30 kDa laminin-binding protein (Figure 4). The type 2 microdomains were also shown to be important for infectivity of alveolar macrophage since after the treatment of yeasts forms with mβ CD, the infectivity was reduced by 45%. It is worth mentioning that infectivity of mβ CD-treated yeasts was completely restored by addition of exogenous ergosterol, but not cholesterol indicating that specifically ergosterol is able to restore the functionality of these fungal membrane domains.

In agreement with these findings, Singh et al. (2012) demonstrated that structural variation in sphingolipids from *C. neoformans* Δ*smt1* mutant strain (which lacks sphingolipid C9 methyltransferase activity) altered the topography of the membrane lipid affecting fungal plasma membrane rigidity, which was associated with a decrease in the fungal pathogenicity. In the same study, they described that sphingolipid microdomains in *C. neoformans* wild type are larger and more tightly packed than in Δ*smt1*. Furthermore, these authors also reported extra “soft areas” in the Δ*smt1* mutant cell membranes, which may lead to a more permeable and more fluid lipid bilayer, resulting in a less rigid conformation of selected sections of the membrane. These data also strongly suggest that the methylated forms of sphingolipids are required for a proper membrane organization associated with fungal virulence.

The dependence of the GSLs organization in plasma membrane for fungal infectivity was studied a using mAbs directed to glycan components of fungal membrane microdomains. We have demonstrated that mAb MEST-3, an IgG2a directed to Manpα3Manpα2IPC, interfered on colony formation and morphological transition from yeast to mycelium of *P. brasiliensis, H. capsulatum* and *S. schenckii*. Similar results were also observed when these fungi where incubated with mAb MEST-1, which reacts with GIPCs presenting terminal residues of β-D-galactofuranose linked to mannose. A possible explanation for these results could be related to the binding of these two mAbs with cell surface GIPCs thus altering the lipid bilayer organization and hindering the formation of functional membrane microdomains leading to interference in GSLs dependent signaling pathways (Toledo et al., 2010).

Membrane microdomains of host cells were also found to be involved in host cell-pathogen interaction. As demonstrated by our group (Maza et al., 2008) in experiments performed with human lung A549 epithelial cells, the membrane rafts of these cells are involved in adhesion process of *P. brasiliensis* yeast forms, promoting activation of Src-family kinases (SFKs) and extracellular signal-regulated kinase 1/2 (ERK1/2) of these epithelial cells. The activation of epithelial cells SFKs or ERK1/2 might be involved in expression of host inflammatory cytokines and therefore *P. brasiliensis* through its microdomains could be involved in modulation of host immune responses.

The concept of “glycosynapses” introduced by Hakomori (2004) helps to understand the nature and complexity of microdomains/membrane rafts interactions between host-fungal cells. The glycan moiety of fungal GIPCs and GlcCer/GalCer acting in a concerted way with carbohydrate sequences of GSLs/glycoproteins of complementary host cell microdomains (*trans*-interaction) may intermediate cell-to-cell adhesion with concurrent signal transduction.

## Conclusions and future perspectives

Due to its importance in clinical contexts, fungal infections have raised questions regarding its biological process and the key molecules related with the infection maintenance and host cell-pathogen interaction. Considering the structural diversity and the biological roles described for fungal GlcCer and GIPCs, disruption of these biosynthetic pathways may represent an interesting/inviting approach to new vistas for fungal infection therapy. Furthermore, considering the influence of fungal GSLs in host immune response, they may also be considered as fungal biomarkers to detect and identify fungal infections, and as well as follow-up markers of a mycosis at different stages.

Taking these findings together, the next steps would include the elucidation of the biological role of GSLs in the interaction of host-fungal cells during the course of the infection. As shown by Maza et al. (2008) and Tagliari et al. (2012) membrane microdomains of both fungi and host cells play important roles in these interactions, such as observed for fungal adhesion to epithelial cells and fungal infectivity of alveolar macrophages. Additionally, other issues regarding the role of fungal cell wall in the cellular contact must also be elucidated. It is tempting to hypothesize that around the fungal membrane microdomain regions, the glycans of the cell wall could be less tight, leading to a more “coarse” organization, this hypothetical model is tentatively shown in Figure 4. This more loose conformation of the cell wall around the microdomain regions could expose these membrane structures such as: adhesins, glycosphingolipids and glycoproteins allowing the cross-talk of pathogen-host cell through the membrane microdomains.

Cellular membranes are not autonomous cellular structures, since they are linked to intracellular and extracellular networks (Nicolson, 2014). One attractive hypothesis is that fungal cell wall could act as biological interface or “conduit” for the information transfer between membrane microdomain and cell wall, perhaps in a glycan-glycan communication and thus mediating the transmission of signals either on localized points or in a network at the cell wall modulating biological events in fungal biology as well as in the fungal infection.

An in-depth knowledge of fungal microdomain interactions in combination with the elucidation of the GSL glycan-dependent signaling pathways and cell wall biosynthesis regulatory steps will certainly provide an integrated view allowing to elaborate a more refined concept of the concerted membrane microdomain-GSL-cell wall involved in key events related to survival and proliferation of pathogenic/opportunistic fungi in the human host.

### Conflict of interest statement

The authors declare that the research was conducted in the absence of any commercial or financial relationships that could be construed as a potential conflict of interest.
